# Insulin Preconditioning Elevates p-Akt and Cardiac Contractility after Reperfusion in the Isolated Ischemic Rat Heart

**DOI:** 10.1155/2014/536510

**Published:** 2014-08-13

**Authors:** Tamaki Sato, Hiroaki Sato, Takeshi Oguchi, Hisashi Fukushima, George Carvalho, Ralph Lattermann, Takashi Matsukawa, Thomas Schricker

**Affiliations:** ^1^Department of Anaesthesia, Royal Victoria Hospital, McGill University Health Center, Montreal, QC, Canada H3A 1A1; ^2^Outcomes Research Consortium, Cleveland, OH 44195, USA; ^3^Department of Anesthesiology, Yamanashi University, Yamanashi 409-3821, Japan

## Abstract

Insulin induces cardioprotection partly via an antiapoptotic effect. However, the optimal timing of insulin administration for the best quality cardioprotection remains unclear. We tested the hypothesis that insulin administered prior to ischemia provides better cardioprotection than insulin administration after ischemia. Isolated rat hearts were prepared using Langendorff method and divided into three groups. The Pre-Ins group (Pre-Ins) received 0.5 U/L insulin prior to 15 min no-flow ischemia for 20 min followed by 20 min of reperfusion. The Post-Ins group (Post-Ins) received 0.5 U/L insulin during the reperfusion period only. The control group (Control) was perfused with KH buffer throughout. The maximum of left ventricular derivative of pressure development (dP/dt(max)) was recorded continuously. Measurements of TNF-*α* and p-Akt in each time point were assayed by ELISA. After reperfusion, dP/dt(max) in Pre-Ins was elevated, compared with Post-Ins at 10 minutes after reperfusion and Control at all-time points. TNF-*α* levels at 5 minutes after reperfusion in the Pre-Ins were lower than the others. After 5 minutes of reperfusion, p-Akt was elevated in Pre-Ins compared with the other groups. Insulin administration prior to ischemia provides better cardioprotection than insulin administration only at reperfusion. TNF-*α* suppression is possibly mediated via p-Akt leading to a reduction in contractile myocardial dysfunction.

## 1. Introduction

In 1986, Murry et al. introduced the concept of ischemic preconditioning (IPC) in which short, repetitive periods of ischemia protected the myocardium from a subsequent, prolonged, and otherwise lethal, ischemic assault [1]. It is also known that the application of certain pharmacological agents to the heart prior to an episode of myocardial ischemia and reperfusion has the capacity to reduce myocardial injury. For example, volatile anesthetics have been shown to induce pharmacological preconditioning (PPC) in a variety of experimental animal models and in humans [2–5].

Insulin is another agent that has been shown to reduce myocardial injury and improve cardiac function, especially when it is combined with normoglycemia [6, 7]. The cardioprotective effect of insulin is induced metabolically by optimizing cardiac metabolism [8, 9], and also nonmetabolically by promoting cardiomyocyte survival pathway [10, 11]. Insulin promotes cardiomyocyte survival by activation of Akt, phosphatidylinositol 3-kinase (PI3K), and p70s6 kinase [10, 12]. The PKB/Akt [13, 14] signaling pathway is implicated in inducing cardioprotection via an antiapoptotic effect [10, 15].

Timing of insulin therapy, related to ischemia, is a crucial factor for its cardioprotective effects. More recently, ischemic postconditioning (IPost) was introduced [16], which can be administered at the time of myocardial reperfusion, and is, therefore, more applicable to situations where myocardial ischemia is already present. The degree of myocardial salvage with IPost has been demonstrated to be comparable with IPC [16], and therefore postconditioning is often a favored strategy because of its applicability. Recent acute myocardial ischemia (AMI) model studies have demonstrated reduction in infarct size in isolated rat heart when insulin was administered after ischemia [17]. On the other hand, our previous studies have shown that high-dose insulin administration prior to cardiopulmonary bypass (CPB) elicits cardioprotection in humans [6, 7]. Insulin improved cardiac contractility when it was administered preemptively before the “iatrogenic ischemia,” caused by CPB. In most of the AMI scenario, conditioning the heart before onset of ischemia is rarely an option. On the contrary, in elective surgery, cardiac conditioning before or after CPB induced ischemia is entirely optional. Nevertheless, the optimal timing of insulin institution for the best quality cardioprotection is still unclear.

In this study, in isolated rat hearts, we tested the hypothesis that insulin administered prior to ischemia provides better cardioprotection than insulin administered after ischemia. The primary measure/outcome was myocardial contractility. We also assessed p-Akt and TNF-*α* in order to identify potential mechanisms underlying the effects of insulin.

## 2. Materials and Methods

### 2.1. Isolated Rat Heart Model

With the approval of the Committee on Animal Research at the Faculty of Medicine, University of Yamanashi, Male Wistar rats (weighing 300–320 g) were anesthetized using intraperitoneal injection of pentobarbital sodium (30 mg/kg body weight).

Their hearts were rapidly excised, immersed in cold saline solution (4°C), and mounted on the stainless-steel cannula of a modified Langendorff perfusion apparatus. Retrograde aortic perfusion was initiated at a perfusion pressure of 55 mm Hg with modified Krebs-Henseleit (KH) buffer solution of the following composition (mmol/liter): NaCl 118, KCl 4.7, CaCl-2H_2_O 2.0, MgSO_4_-7H_2_O 1.2, KH_2_PO_4_ 1.2, glucose 5.5 (99 mg/dL), and NaHCO_3_ 25. The perfusate was bubbled continuously with 95% O_2_ and 5% CO_2_ and maintained at 37°C 1hroughout the experiment. A water-filled latex balloon containing a pressure transducer (TruWave, Edwards Lifesciences, CA, USA) was placed into the left ventricle (LV) through the left atrium to measure LV function. The heart was paced to 222 beats/min during the ischemic period with electronic stimulator (SEN-3201, Nihon Kohden, Tokyo, Japan).

### 2.2. Experimental Protocol

**Figure 1:**
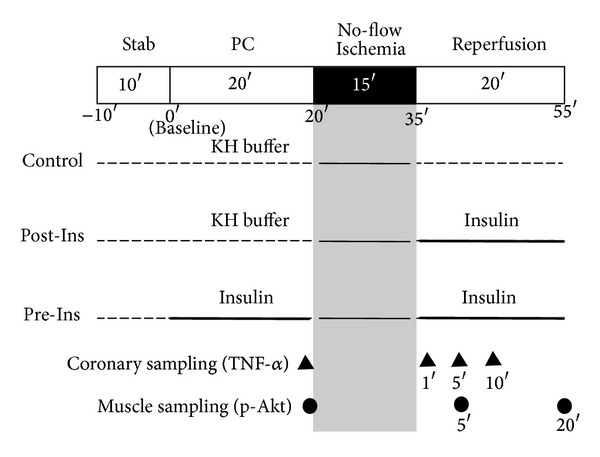
Experimental protocol. The Pre-Ins group received 0.5 U/L insulin in KH buffer prior to 15 min no-flow ischemia for 20 min followed by 20 min of reperfusion. The Post-Ins group received 0.5 U/L insulin during the reperfusion period only. The control group was perfused with KH buffer throughout.

The animals were divided into three groups and the experimental protocols are shown in Figure 1. The Pre-Ins group received 0.5 U/L insulin in KH buffer prior to 15 min no-flow ischemia for 20 min and during 20 min of reperfusion. The Post-Ins group received 0.5 U/L insulin during the reperfusion period only. The control group was perfused with KH buffer throughout.

### 2.3. Measurements

Heart rate (bpm) and maximum left ventricular derivative of pressure development (LV dP/dt max) (mmHg/sec) were recorded continuously. Coronary flow (mL/min) was measured by timed collection of the perfusate (baseline, after 20 min of preconditioning, and after 5, 10, 15, and 20 minutes of reperfusion).

Coronary efferent fluid samples for TNF-*α* measurements were drawn after 20 min of preconditioning and after 1, 5, and 10 minutes of reperfusion. The concentrations of TNF-*α* were measured by the sandwich ELISA technique (Invitrogen rat TNF-*α* ELISA Kit, Life Technologies, CA, USA) and quantified photometrically (Spectra Max 340, Molecular Devices, CA, USA) at an absorbance of 450 nm. The values were expressed as pg/mL. Measurements of TNF-*α* in the coronary effluent were normalized to 1 min volume of coronary flow.

At the end of the perfusion (after 20 min of reperfusion), the whole heart was quickly frozen in liquid nitrogen and freeze-dried for six days. The myocardium was suspended in assay lysis buffer (Lysis Buffer 6, R&D Systems, MN, USA) containing phenylmethanesulfonyl fluoride (PMSF, 2 mM, Sigma-Aldrich, Inc., MO, USA) and protease inhibitor cocktail (Sigma-Aldrich, Inc., MO, USA). The samples were then homogenized using a microhomogenizing system (MicroSmash MS-100R, TOMY SEIKO Co., Ltd., Japan). The homogenates were centrifuged for 5 min at 2000 g and the supernatants were assayed for their p-Akt content by ELISA (Surveyor IC Human/Mouse/Rat Phospho-Akt (Pan) (S473) Immunoassay, R&D Systems, MN, USA). At the other time points (after 20 min of preconditioning and after 5 min of reperfusion), muscle samples of whole heart for p-Akt measurements were taken using different rats (*n* = 12 in each group) in the above same technique (Figure 1). The concentrations of p-Akt (ng/gram of dry heart weight) were measured by the sandwich ELISA technique and quantified photometrically (Spectra Max 340, Molecular Devices, CA, USA) at an absorbance of 450 nm. The values were expressed as ng of p-Akt per gram of dry heart weight.

### 2.4. Statistical Analysis

The data are presented as means ± SD. Changes in hemodynamics and the concentrations of TNF-*α* and p-Akt were analyzed using two-way analysis of variance (ANOVA), followed by the Bonferroni post hoc test. Intergroup comparisons for baseline measurements, hemodynamic data, and the concentrations of TNF-*α* and p-Akt at each time point were made with one-way ANOVA followed by the Bonferroni post hoc test. Two-sided *P* values less than 0.05 were considered statistically significant.

Sample size calculation was based on the expected difference in the contractility (LV dP/dt max) at 20 min after reperfusion among groups. The results of a preliminary of our pilot study showed contractility of 1000 ± 800 (mmHg/sec) in the control group, 1500 ± 1000 in the Post-In group, and 3000 ± 1500 in the Pre-In group at 20 min after reperfusion. In order to achieve a power level of 80%, with an alpha error of 5%, at least 12 subjects were required in each group. All statistical analyses were performed using SPSS 21 for Windows (IBM, Chicago, IL) and PASS 11 (NCSS, Kaysville, UT).

## 3. Results and Discussion

There was no significant difference in baseline measurements between the groups (Table 1).

**Figure 2:**
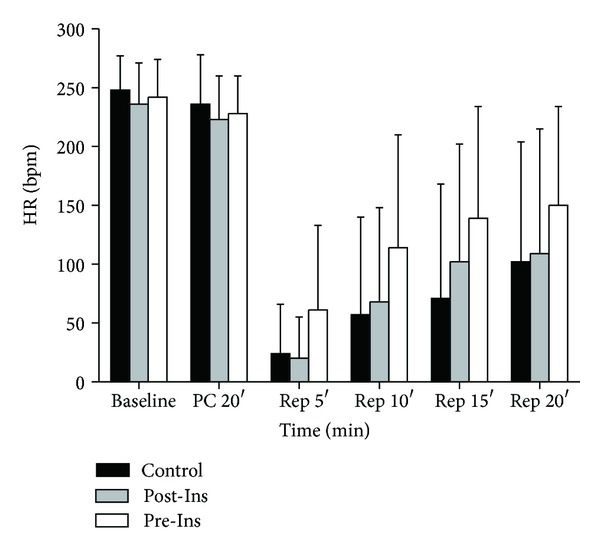
Time-course changes of heart rate before and after ischemia in the three groups (*n* = 12 in each group). The data are presented as means ± SD. HR: heart rate (bpm).

**Figure 3:**
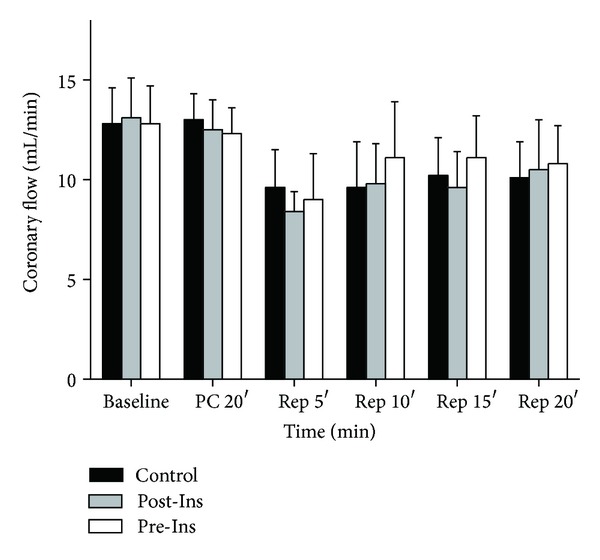
Time-course changes of coronary flow before and after ischemia in the three groups (*n* = 12 in each group). Coronary flow (mL/min) was measured by timed collection of the perfusate (baseline, after 20 min of preconditioning and after 5, 10, 15, and 20 minutes of reperfusion). The data are presented as means ± SD.

Heart rate increased gradually after reperfusion in all groups (Figure 2); however there were no differences between groups. Coronary flow was comparable among all experimental groups (*P* = 0.58) (Figure 3).

**Figure 4:**
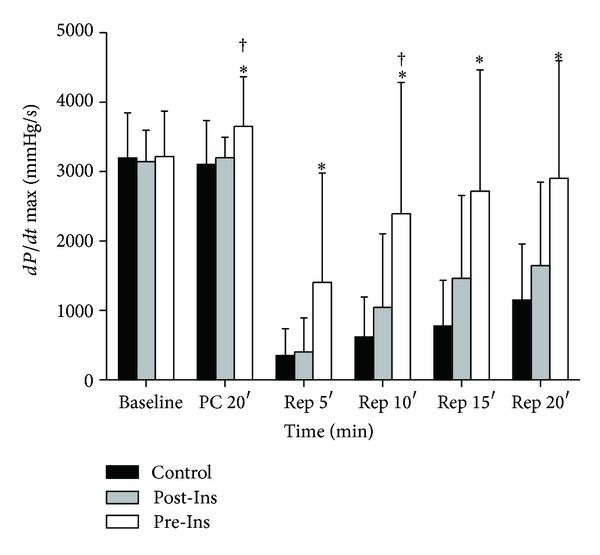
Time-course changes of LV dP/dt max before and after ischemia in the three groups (*n* = 12 in each group). The data are presented as means ± SD. *P < 0.05 versus Control; ^†^ *P* < 0.05 versus Post-Ins. In Pre-Ins, LV dP/dt max increased after administration of insulin prior to ischemia (PC 20′) compared to both Post-Ins and Control. After reperfusion, dP/dt max in Pre-Ins was elevated, compared with Post-Ins at 10 minutes after reperfusion (Rep 10′) and Control at all-time points (Rep 5′, 10′, 15′, and 20′). dP/dt max (mmHg/sec): maximum of left ventricular derivative of pressure development.

In Pre-Ins group, LV dP/dt max increased after administration of insulin prior to ischemia (after 20 min of preconditioning) compared to both Post-Ins and control groups (Figure 4). After reperfusion, LV dP/dt max in Pre-Ins group was significantly elevated, compared with Post-Ins groups at 10 minutes after reperfusion and control groups at all-time points (after 5, 10, 15, and 20 minutes of reperfusion).

**Figure 5:**
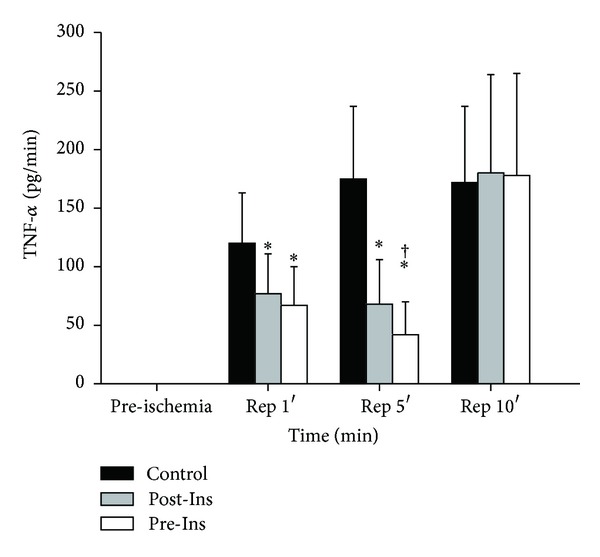
Time-course changes of TNF-*α* before and after ischemia in the three groups (*n* = 12 in each group). Coronary efferent fluid samples for TNF-*α* measurements were drawn after 20 min of preconditioning (preischemia) and after 1, 5, and 10 minutes of reperfusion. Measurements of TNF-*α* in the coronary effluent were normalized to 1 min volume of coronary flow (pg/mL). The data are presented as means ± SD. *P < 0.05 versus Control; ^†^ *P* < 0.05 versus Post-Ins. A significant increase in TNF-*α* was observed in Control at the beginning of reperfusion (Rep 1′ and 5′). The TNF-*α* at 5 minutes after reperfusion (Rep 5′) in the Pre-Ins was lower than both Post-Ins and Control.

The TNF-*α* concentrations, which were normalized to 1 min volume of coronary flow, were below the detectible range in all groups before ischemia. A significant increase in TNF-*α* level was observed in the control group at the beginning of reperfusion (Figure 5). Independent of timing, insulin attenuated the increase in TNF-*α* at 1 and 5 minutes after reperfusion. The TNF-*α* levels at 5 minutes after reperfusion in the Pre-Ins group were significantly lower than both Post-Ins and control groups.

**Figure 6:**
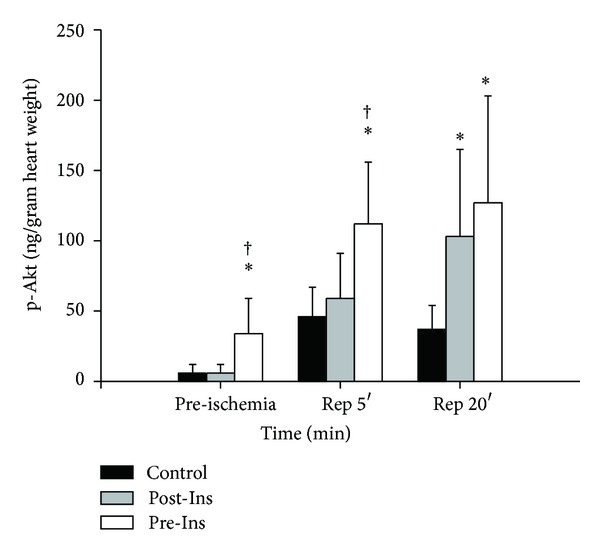
Time-course changes of p-Akt before and after ischemia in the three groups. Muscle samples for p-Akt concentrations (ng/gram of dry heart weight) were taken after 20 min of preconditioning (preischemia, *n* = 12 in each group) and after 5 min of reperfusion (*n* = 12 in each group) and 20 min of reperfusion (*n* = 12 in each group). The data are presented as means ± SD. *P < 0.05 versus Control; ^†^ *P* < 0.05 versus Post-Ins. In Pre-Ins, p-Akt increased after administration of insulin before ischemia (preischemia). After 5 minutes of reperfusion, p-Akt was elevated in the Pre-Ins when compared with Control and Post-Ins (Rep 5′). The p-Akt levels in the Pre-Ins were still higher than Control after 20 minutes of reperfusion (Rep 20′).

The levels of p-Akt increased after administration of insulin before ischemia (Figure 6). After 5 minutes of reperfusion, p-Akt was elevated in the Pre-Ins group when compared with values observed in the control and Post-Ins group. The p-Akt levels in the Pre-Ins group were still significantly higher than in Control after 20 minutes of reperfusion.

In present study, insulin administration prior to ischemia provides better cardiac contractility than insulin administered only at reperfusion. The myocardium p-Akt was increased and maintained at the highest level in the Pre-Ins group, where suppression of TNF-*α* and elevated cardiac contractility were also observed. This increase in p-Akt resulted in improved cardiac function at reperfusion suggesting that TNF-*α* suppression is possibly mediated via p-Akt leading to a reduction in contractile myocardial dysfunction.

The Akt plays an important role in IPC protecting the myocardium from prolonged ischemia [1, 18]. Targeting proteins in IPC induced prosurvival pathway, such as p-Akt, with pharmacological agents is shown to provide similar cardioprotection, which is termed “pharmacological pre- or postconditioning.” Insulin is well known to activate the Akt signaling cascade with various effects. In the current study, insulin in the Pre-Ins group elevated p-Akt in cardiomyocytes prior to ischemia compared with the noninsulin perfused heart and was maintained at the highest level during reperfusion. It is also known that ischemia itself activates the cell survival p-Akt cascade [19], and all three groups showed increases in p-Akt above baseline after ischemia. The prosurvival kinase Akt is the common signaling cascade that both insulin and IPC share in reducing cardiac ischemia-reperfusion injury [20] and “conditioning” the heart. Applying both insulin and ischemia amplifies the degree of Akt phosphorylation as shown in Pre- and Post-Ins group and possibly augments the cardioprotective conditioning effect.

Although essential for tissue survival, oxygen can be injurious during reperfusion. The intracellular changes during ischemia and reperfusion lead to the formation of reactive oxygen species (ROS), which play an important role in ischemia-reperfusion injury [21]. The cytokine-mediated cascade is initiated by this nonspecific injury, which results in the production of TNF-*α* [22]. Excessive TNF-*α* expression directly induces contractile myocardial dysfunction and cell apoptosis [23]. The current study demonstrates that TNF-*α* was suppressed in Pre- and Post-Ins group, especially in the Pre-Ins group at 5-minute reperfusion. It has been suggested that TNF-*α* inhibition is partly regulated by the p-Akt pathway [24]. Thus, one may speculate that insulin infusion increased p-Akt and promoted TNF-*α* suppression, which then resulted in preserved cardiac contractility in Pre-Ins group.

Several studies emphasize the importance of the timing of insulin in relation to myocardial ischemia [10, 25]. It is not yet clear, however, whether preconditioning the heart with insulin provides the best quality protection or delayed insulin administration, that is, postconditioning, is equally effective. The current study results show an increase in p-Akt, TNF-*α* suppression, and a better preserved LV dP/dt max, when insulin was administered before ischemia compared to when it was started later. The results suggest that insulin preconditioning provides better cardioprotection than postconditioning in ischemia-reperfusion injury. Interestingly, differences in p-Akt and TNF-*α* between Pre- and Post-Ins group are no longer significant at 20 minutes of reperfusion, where LV dp/dt remains the highest in Pre-Ins group. This can be explained by the possible existence of a threshold of Akt activation which is required to confer cardioprotective effect [26, 27]. Applying insulin prior to ischemia activates the cell survival p-Akt cascade early enough to protect the myocardium during reperfusion.

Recent studies demonstrated that the cardioprotective effect of insulin against myocardial infarction occurred when insulin was administered only during reperfusion, but not when started prior to ischemia [9, 10]. Jonnasen et al. studied isolated rat hearts applying 35 minutes regional myocardial ischemia. They showed that the administration of insulin at a high dose, for 10 minutes prior to ischemia, continued throughout ischemia and reperfusion, failed to reduce infarct size. Interestingly, administration of insulin only at the onset of reperfusion significantly reduced infarct size [10]. On the other hand, our current study showed that administration of insulin prior to ischemia preserved cardiac contractility better than insulin administration only during reperfusion. Discrepancies in cardioprotection related to the timing of insulin administration may be explained by differences in study protocol and measurements. Jonnasen's study measured infarct size with regional myocardial ischemia, whereas the present study assessed whole heart ischemia and cardiac function. They have suggested that insulin inhibits proapoptotic protein on outer mitochondrial membrane which resulted in reduction of infarct size, whereas in our study, insulin via pAkt inhibits TNF-*α* production putatively and thus also inhibits TNF-*α* effect directly on myocardial contractile dysfunction [28, 29]. The inotropic effect of insulin, while the myocardium is exposed to regional ischemia, possibly attenuates cell survival by aggravating the imbalance between myocardial oxygen supply and demand.

## 4. Conclusions

In conclusion, administration of insulin prior to ischemia protected cardiac contractility better than insulin administered during reperfusion. The p-Akt activity in cardiomyocyte was significantly higher when insulin was administered before ischemia supporting the contention that the p-Akt cell survival pathway contributes to “conditioning” effect of IPC and PPC. The current study suggests that insulin activated p-Akt also facilitates preservation of cardiac contractility during ischemia-reperfusion injury in the isolated rat heart.

## Figures and Tables

**Table 1:** Baseline measurements.

	Control	Post-Ins	Pre-Ins
Number (*n*)	12	12	12
Rat weight (g)	303 ± 5	301 ± 3	303 ± 5
Dry heart weight (g)	0.24 ± 0.05	0.23 ± 0.02	0.23 ± 0.04
Heart rate (bpm)	248 ± 29	236 ± 35	242 ± 32
dP/dt max (mm Hg/sec)	3197 ± 648	3141 ± 453	3215 ± 653
Coronary flow (mL/min)	12.8 ± 1.8	13.1 ± 2.0	12.8 ± 1.9

Data are mean ± SD. There are no significant differences among the groups by analysis of variance.

Baseline measurements are presented in absolute values as obtained after 10 min stabilization except for dry heart weight which was measured at the end of the experiment.

dP/dt max: maximum of left ventricular derivative of pressure development.
